# Budd-Chiari Syndrome Secondary to Essential Thrombocythaemia Complicated by Acquired Von Willebrand Disease and Mimicking Hepatic Malignancy: A Case Report

**DOI:** 10.7759/cureus.100145

**Published:** 2025-12-26

**Authors:** Faryal Rahim, Khadija Ali, Yen Yi Lee, Hans Siy-yap, Shenaz Joomye

**Affiliations:** 1 Internal Medicine, Manchester University NHS Foundation Trust, Manchester, GBR; 2 Emergency Medicine, North Manchester General Hospital, Manchester, GBR; 3 Diabetes and Endocrinology, Mersey and West Lancashire Teaching Hospitals NHS Trust, Prescot, GBR; 4 Internal Medicine, North Manchester General Hospital, Manchester, GBR

**Keywords:** acquired von willebrand disease, budd-chiari syndrome, case report, essential thrombocythemia, hepatic vein stenting, jak2 gene mutation

## Abstract

Budd-Chiari syndrome (BCS) secondary to essential thrombocythaemia (ET) is an uncommon but clinically significant complication of myeloproliferative neoplasms (MPNs). This report presents a case of a male in his mid-40s who presented with deranged liver function tests (LFTs), dyspnoea, fatigue, pruritus, and abdominal distension. Initial CT imaging suggested a possible hepatic malignancy; however, subsequent MRI demonstrated features strongly indicative of BCS. Further investigations confirmed Janus kinase 2 (JAK2) mutation-positive ET and acquired von Willebrand syndrome (AvWS). The patient was managed with cytoreductive therapy; initially, hydroxycarbamide and anagrelide, later transitioned to ruxolitinib, alongside lifelong anticoagulation. He later underwent successful hepatic vein recanalization and venoplasty. At six-month follow-up, he demonstrated improved LFTs, stable blood counts, and no recurrent thrombotic events, with ongoing surveillance for variceal bleeding. This case underscores the importance of considering BCS in patients with ET and hepatic abnormalities, screening for AvWS to balance thrombotic/bleeding risks, and utilizing a multidisciplinary team (MDT) approach for optimal management.

## Introduction

Budd-Chiari syndrome (BCS) is a rare, but potentially fatal, syndrome with an estimated incidence of 0.1-10 per million per year in the UK [1]. It is characterized by hepatic venous outflow obstruction at the level of the hepatic vein or inferior vena cava, causing portal hypertension. Hepatic congestion and hypoxic injury result in centrilobular necrosis and fibrosis of the liver [2]. BCS classically presents with the triad of abdominal pain, hepatomegaly, and ascites [1]. Untreated BCS has a poor prognosis, with reported mortality approaching 90% within three years in the UK. Most patients die within three months to three years from the time of diagnosis due to complications of portal hypertension, hepatic encephalopathy, and variceal haemorrhage [3]. Hypercoagulable states are the leading cause of BCS in the West [4]. Prompt diagnosis and management of BCS in its early stages is paramount to avoid complications from developing.

The diagnostic challenges of BCS largely arise from its rarity and the broad, non-specific nature of its clinical presentation. Symptoms frequently overlap with other hepatic conditions, such as cirrhosis or drug-induced hepatic injury, leading to frequent delayed or misdiagnosis. In chronic forms, patients may present with minimal or even absent symptoms and normal or near-normal liver function tests (LFTs), further obscuring recognition, although our patient presented with significant cholestasis, highlighting the variable nature of the syndrome [5]. Additionally, in cases associated with essential thrombocythaemia (ET), BCS can clinically and radiologically mimic hepatic malignancy or metastatic disease, contributing to its diagnostic complexity [6]. Although the classic triad of abdominal pain, ascites, and hepatomegaly present in BCS is more commonly observed in acute presentations, these features are also non-specific and shared with other common hepatic and cardiac disorders [1]. Therefore, a high index of clinical suspicion is essential to ensure timely evaluation and management.

ET, caused by the clonal proliferation of atypical megakaryocytes, is a rare chronic myeloproliferative disorder (MPD) resulting in extreme thrombocytosis (>1000 x 10^9^/L). It has an estimated annual incidence of approximately 2.2 per 100,000 individuals in the UK, typically presenting at a median age of 65 years, with a female predominance [7,8]. ET is most commonly associated with one of three driver mutations: Janus kinase 2 (JAK2), calreticulin (CALR), or myeloproliferative leukaemia virus oncogene (MPL) [8]. Among these, the JAK2 mutation is the most prevalent and is associated with a comparatively higher risk of thrombotic complications than CALR or MPL-mutated disease [9]. The patient described in this case report had JAK2-positive ET. JAK2-positive ET predisposes patients to a higher risk of venous thrombus formation, including involvement of the hepatic veins. While hepatic complications may occur across several myeloproliferative neoplasms (MPNs), multiple studies have established ET as the leading MPN cause of BCS [4].

ET is also associated with an increased bleeding risk due to the development of acquired von Willebrand syndrome (AvWS). AvWS is a rare, non-inherited disorder that clinically resembles inherited von Willebrand disease (vWD) [10]. Although classically linked to lymphoproliferative and cardiovascular disorders, emerging evidence indicates that AvWS is also seen in MPNs. In ET, markedly elevated platelet counts (>1000 x 10^9^/L) enhance the interaction between glycoprotein Ib (GP1b) receptors and von Willebrand factor (vWF), leading to accelerated clearance of vWF from the circulation [11,12]. AvWS is diagnosed by demonstrating reduced vWF activity-to-antigen ratios and a loss of high-molecular-weight vWF multimers on gel electrophoresis, alongside a clinical picture of a tendency to bleed [12]. This bleeding risk is further exacerbated when anticoagulation is required for the management of ET. This dual risk of thrombosis and bleeding creates significant clinical challenges, requiring careful individualized risk assessment and treatment decisions.

## Case presentation

A middle-aged man was referred by his general practitioner due to deranged LFTs and a nine-month history of progressive abdominal distension, bilateral lower limb swelling, fatigue, pruritus, and exertional dyspnoea. He had no past medical history and no family history of note. He had a history of travel to West Africa, which coincided with the onset of symptoms. He reported no history of alcohol consumption or smoking. On physical examination, he was noted to have mildly icteric sclerae and koilonychia. Cardiovascular examination revealed a systolic murmur. Abdominal examination demonstrated distension with mild central tenderness, and bilateral pitting oedema was present up to the knees.

Initial laboratory investigations (Table 1) showed severe microcytic anaemia (haemoglobin = 68 g/L, mean corpuscular volume = 53 fL), marked thrombocytosis (platelets = 1074 ×10^9/L), and elevated white cell count (17 ×10^9/L) with neutrophilia and mild eosinophilia. Liver function tests revealed a cholestatic pattern with elevated alkaline phosphatase (412 U/L), alanine transaminase (108 U/L), gamma-glutamyl transferase (607 U/L), mildly elevated bilirubin (42 µmol/L), and hypoalbuminemia (32 g/L). Coagulation studies were within normal limits, and tumour marker alpha-fetoprotein was negative. Iron studies confirmed iron deficiency (serum iron = 10 µmol/L, ferritin = 6 µg/L, transferrin saturation = 14.4%) with elevated total iron-binding capacity (94 µmol/L). Blood film demonstrated teardrop cells, platelet anisocytosis, and polychromasia.

**Table 1:** Laboratory test results showing haematology, iron studies, liver function, coagulation, and tumour markers. Abnormal values indicate iron deficiency anaemia with reactive thrombocytosis and evidence of liver dysfunction. Hb: haemoglobin; MCV: mean cell volume; WCC: white cell count; TIBC: total-iron binding capacity; CRP: C-reactive protein; ALP: alkaline phosphatase; ALT: alanine transaminase; GGT: gamma-glutamyl transferase; PT: prothrombin time; APTT: activated partial thromboplastin time; AFP: alpha-fetoprotein; g/L: grams per litre; fL: femtolitres; ×10⁹/L: billion per litre; µmol/L: micromoles per litre; µg/L: microgram per litre; mg/L: milligrams per litre; U/L: units per litre; g/L: grams per litre; sec: seconds; ng/mL: nanograms per millilitre. An adult is defined as ≥16 years of age. ♀ female, ♂ male symbol.

Test	Result	Unit	Normal UK range (adult)	Comment/Interpretation
Haematology				
Hb	68	g/L	♀ 115–155 g/L; ♂ 130–170 g/L	Low – indicates anaemia
MCV	53	fL	80–100 fL	Low – microcytic anaemia
Platelet	1074	×10⁹/L	150–400 ×10⁹/L	High – thrombocytosis
WCC	17.0	×10⁹/L	4.0–11.0 ×10⁹/L	Elevated
Neutrophils	11.99	×10⁹/L	2.0–7.5 ×10⁹/L	High
Eosinophils	0.90	×10⁹/L	0.0–0.5 ×10⁹/L	Mildly elevated
Blood film	-	-	—	Iron deficiency anaemia with occasional teardrops, platelet anisocytosis, polychromasia
Iron studies				
Serum iron	10	µmol/L	10–30 µmol/L	Low
Ferritin	6	µg/L	♀ 11–310 µg/L; ♂ 24–340 µg/L	Low – confirms iron deficiency
TIBC	94	µmol/L	45–72 µmol/L	Usually elevated in iron deficiency
Transferrin saturation	14.4	%	20–45%	Low
Inflammation				
CRP	11	mg/L	<5 mg/L	Mildly elevated
Liver function				
Bilirubin	42	µmol/L	3–20 µmol/L	Mildly elevated
ALP	412	U/L	30–130 U/L	Elevated
ALT	108	U/L	<41 U/L	Elevated
GGT	607	U/L	<60 U/L	Elevated
Albumin	32	g/L	35–50 g/L	Low
Coagulation				
PT	12.3	sec	11–15 sec	Normal
APTT	27.5	sec	25–36 sec	Normal
Tumour marker				
AFP	<3	ng/mL	< 10 ng/mL	Negative

**Figure 1:**
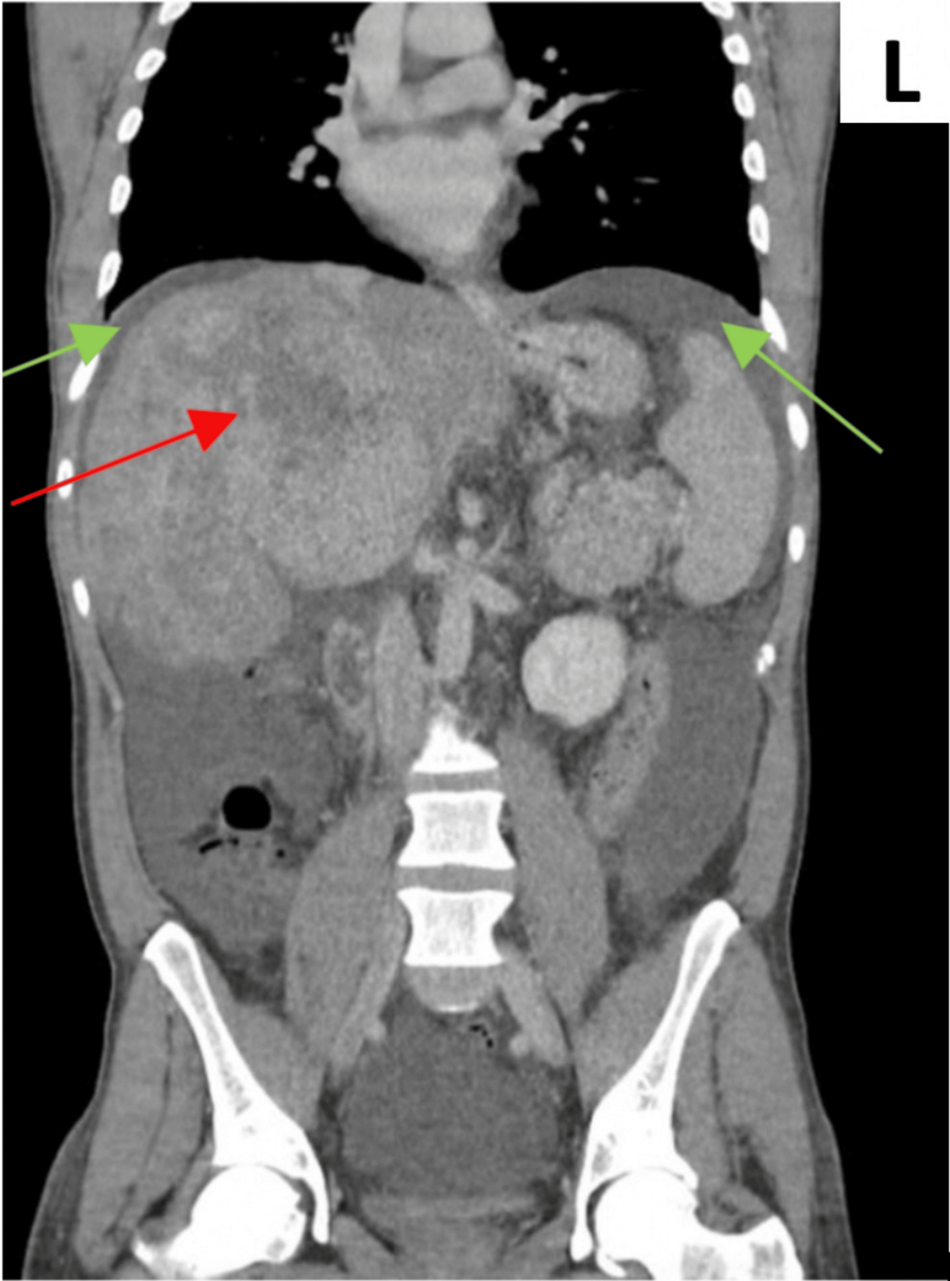
Coronal plane from a contrast-enhanced CT of the thorax, abdomen, and pelvis. The red arrow indicates a large, ill-defined mass occupying the right hepatic lobe, consistent with a possible malignant lesion. Green arrows highlight small bilateral pleural effusions. Although not shown in these images, additional findings included low-density cystic lesions within the spleen and the presence of ascites. No enlarged abdominal or pelvic lymph nodes were identified. ‘L’ indicates the left side of the body.

A wide range of differential diagnoses was considered, given the examination findings and investigation results, such as first presentation of decompensated liver disease, spontaneous bacterial peritonitis (SBP), parasitic infection, and malignancy. An ascitic tap was performed; however, it was unsuccessful due to non-significant ascites. A non-invasive liver screen was performed and ruled out alternative aetiologies, including infectious hepatitis, haemochromatosis, Wilson’s disease, alpha-1 antitrypsin deficiency, and parasitic serologies, including schistosomiasis, filaria, hydatid, and strongyloidiasis (these were all negative), supporting attribution of the eosinophilia to the underlying MPN. He was commenced on broad-spectrum antibiotics and had urine and blood samples sent for microscopy, culture, and sensitivities. His inflammatory markers subsequently improved. CT imaging (Figure 1) revealed a suspicious lesion in the right lobe of the liver. Biliary atresia was ruled out on imaging. Following review at the hepatobiliary multidisciplinary team (HPB MDT) meeting, the findings were initially interpreted as suggestive of advanced hepatocellular carcinoma.

**Figure 2:**
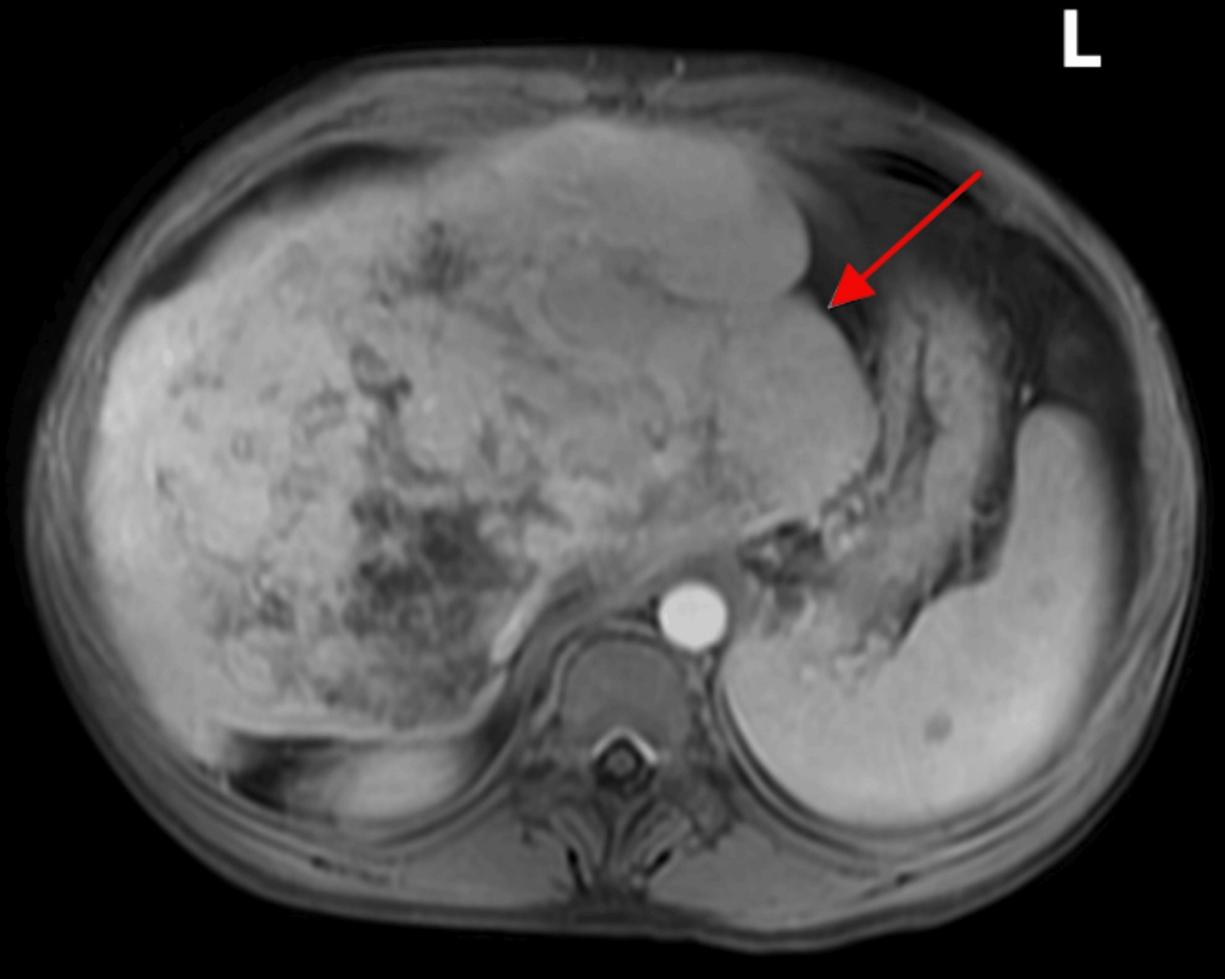
Axial MRI (T1-weighted water image) of the upper abdomen performed with gadolinium contrast, showing marked hepatomegaly with heterogeneous signal intensity throughout the liver. The red arrow points to the markedly enlarged caudate lobe of the liver, a characteristic finding in chronic Budd-Chiari syndrome. The central liver parenchyma shows architectural distortion and progressive contrast enhancement, consistent with fibrosis and venous outflow obstruction. No focal discernible liver lesion was identified on the MRI to suggest malignancy. ‘L’ indicates the left side of the body.

However, subsequent MRI (Figure 2), done to further evaluate the lesion, demonstrated a large heterogeneous area in the right liver lobe but no discrete mass; features were consistent with chronic BCS with associated portal vein thrombosis. The case was re-discussed at the HPB MDT, and a consensus diagnosis of BCS secondary to hepatic and portal vein thrombosis was reached.

**Figure 3:**
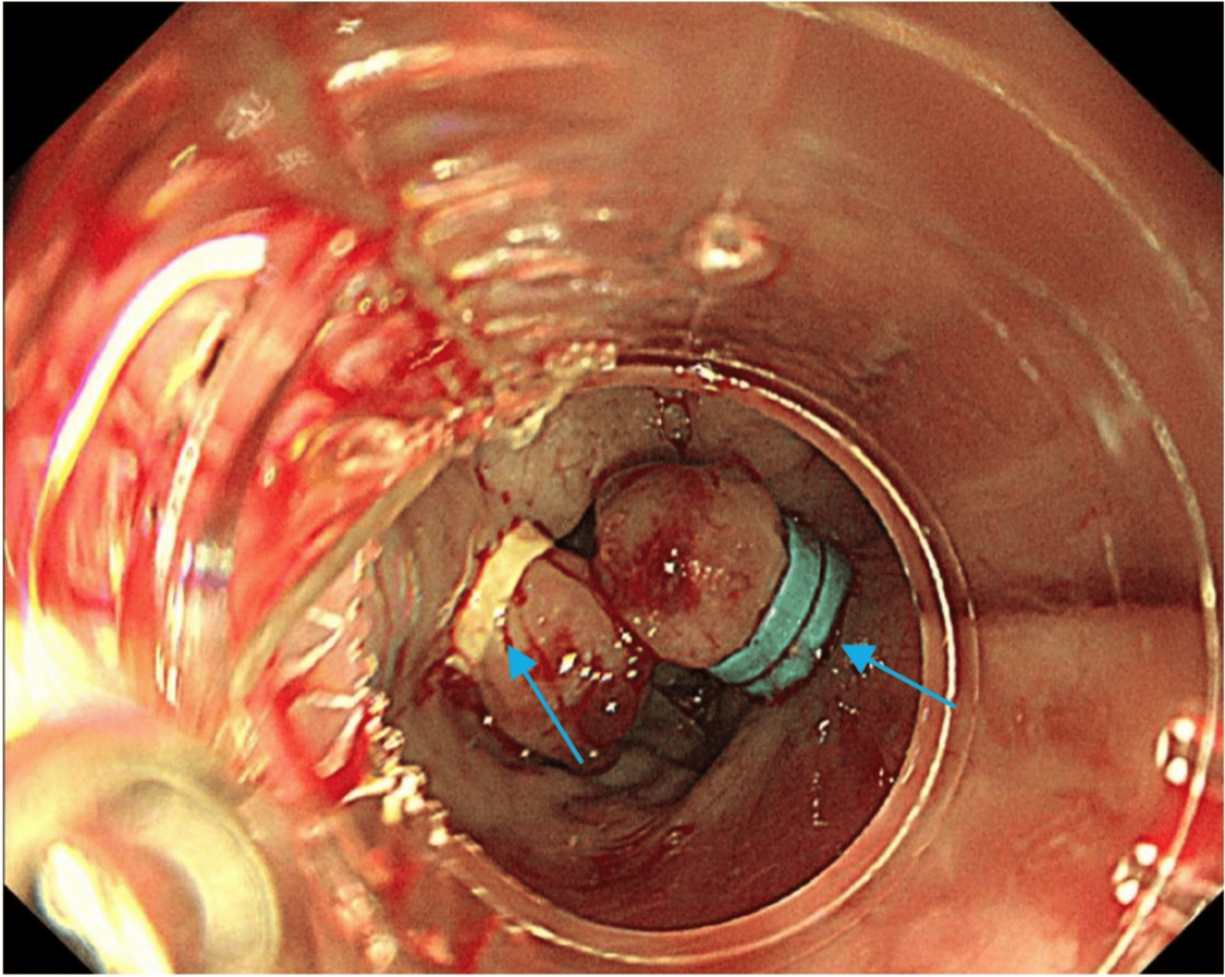
Oesophagogastroduodenoscopy showing active bleeding, with varices showing signs of stigmata of recent haemorrhage. Endoscopic variceal band ligation has been performed, with visible rubber bands (marked by the blue arrow) placed around the varices to achieve haemostasis. These findings are consistent with portal hypertension, commonly seen in chronic Budd-Chiari syndrome and other causes of hepatic decompensation.

Despite IV iron replacement and resolution of infection with antibiotics, the marked thrombocytosis persisted and progressively worsened, prompting evaluation for primary causes. Peripheral blood film findings reinforced suspicion for an MPN. Comprehensive haematology testing revealed JAK2 mutation-positive ET. Further diagnostic workup revealed low vWF ristocetin cofactor (vW: RiCoF) activity and decreased vWF collagen-binding activity (vWF: CB), with normal vWF antigen and factor VIII levels, indicating a qualitative vWF defect and confirming a diagnosis of AvWS, consistent with the patient’s clinical picture of recurrent variceal bleeding. Oesophagogastroduodenoscopy (OGD) and band ligation were performed to stop the variceal bleeding (Figure 3).

The patient was commenced on cytoreductive therapy aimed at lowering the platelet count and mitigating endothelial activation as part of ET management. He was commenced on hydroxycarbamide and anagrelide; however, anagrelide was discontinued due to a rapid decline in platelet count to <1000 ×10⁹/L, and the hydroxycarbamide dose was initially reduced. On subsequent up-titration of hydroxycarbamide, the platelet count further fell to 18 ×10⁹/L. In view of the marked platelet lability under specialist haematology input, the patient was bridged to and commenced on ruxolitinib. Hydroxycarbamide was subsequently discontinued, and ruxolitinib continued under close haematology supervision. The platelet count subsequently stabilized.

**Figure 4:**
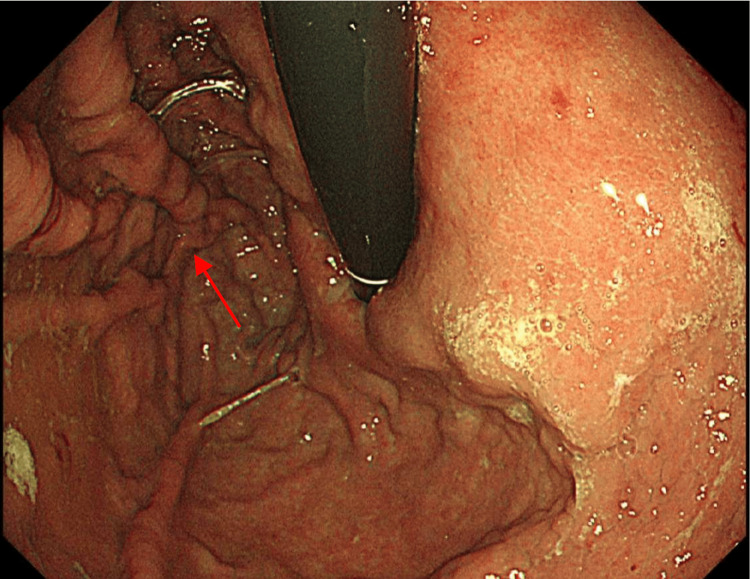
Oesophagogastroduodenoscopy image demonstrating extensive oesophageal varices (marked by the red arrow) occupying the distal oesophagus.

To manage the concurrent thrombotic risk, the patient was initially commenced on low-molecular-weight heparin (LMWH); however, this was discontinued following a decline in platelet count and recurrent variceal bleeding. After resolution of the bleeding, prophylactic-dose LMWH was reintroduced while platelet counts remained labile. The patient underwent multiple repeat endoscopies, revealing grade II and III oesophageal varices (Figure 4), which were treated with aggressive prophylactic variceal band ligation to minimize further bleeding risk. Peri-procedural bleeding risk during OGD was managed by holding the prophylactic-dose LMWH prior to the procedure and administering red cell concentrate transfusion and terlipressin. Once platelet counts stabilized, LMWH was increased to therapeutic dosing under close haematology follow-up. After two weeks of stable platelet counts and vW: RiCoF levels, anticoagulation was transitioned to apixaban, a direct oral anticoagulant.

The right hepatic vein was successfully recanalized using venoplasty by interventional radiology, restoring adequate hepatic venous outflow. Specialist haematology review, including bone marrow biopsy, demonstrated pre-fibrotic changes; however, cytogenetic and myeloid panel testing were negative aside from the JAK2 mutation.

Clinical review at six months showed improving LFTs, no side effects to ruxolitinib, stable platelet counts, and no further thrombotic events, while monitoring for variceal bleeds continued.

## Discussion

This case describes a middle-aged man who developed BCS secondary to JAK2-positive ET, complicated by AvWS, variceal bleeding, and portal hypertension. It highlights the diagnostic challenges of BCS and the complexity of managing the "MPN paradox", the concurrent thrombosis, and bleeding (from AvWS) risk in the same patient.

BCS is characterized by hepatic venous outflow obstruction and is classically associated with the triad of ascites, hepatomegaly, and abdominal pain [2]. However, the initial presentation is heterogeneous and often reflects the underlying aetiology. BCS is most frequently linked to prothrombotic conditions, particularly MPNs such as polycythaemia vera (PV) and ET. Notably, JAK2-positive MPNs are identified in approximately 50% of patients with primary BCS [13]. The JAK2 mutation not only drives clonal myeloid proliferation, leading to increased platelet production and activation, but is also expressed in circulating endothelial progenitor cells, inducing aberrant endothelial behaviour through upregulation of adhesion molecules and the creation of a pro-inflammatory, pro-coagulant vascular surface [14]. This chronic endothelial dysfunction, coupled with heightened platelet reactivity, promotes local thrombus formation within the hepatic venous system, where altered flow dynamics further predispose to stasis and thrombosis, forming Virchow’s triad [13,14]. Other inherited and acquired thrombophilic disorders also confer risk, including factor V Leiden mutation, prothrombin gene mutation, protein C or S deficiency, antiphospholipid syndrome, and pregnancy-associated thrombosis [15].

Autoimmune and infectious conditions have also been implicated. For instance, a case has been reported of a 32-year-old female who presented with progressive abdominal distension over four months and was subsequently diagnosed with BCS secondary to systemic lupus erythematosus (SLE) [16]. More recently, the prothrombotic state associated with COVID-19 has become increasingly recognized. One report described a 48-year-old woman who presented with BCS in the absence of any known underlying risk factors and was later found to have COVID-19 infection, highlighting the diverse and multifactorial nature of BCS [17]. Hence, a prompt investigation to identify the underlying cause is essential.

Timely diagnosis and management of BCS require a coordinated multidisciplinary approach involving hepatology, haematology, radiology, and interventional specialists. Early identification of the underlying cause and prompt initiation of treatment are associated with significantly improved outcomes; the five-year survival rate of treated BCS approximates 90% [18].

Management follows a stepwise strategy. Anticoagulation forms the cornerstone of therapy, aiming to prevent further thrombus propagation, allow recanalization, and reduce hepatic venous congestion. This helps to limit ongoing hepatocellular injury and supports hepatic regeneration [2]. Where obstruction involves a short segment of the hepatic veins or inferior vena cava, endovascular interventions such as angioplasty or stent placement may be beneficial [19]. In cases of persistent portal hypertension or inadequate response to anticoagulation and angioplasty, a transjugular intrahepatic portosystemic shunt (TIPS) can effectively decompress the portal system and improve hepatic function [20]. For patients with refractory disease, surgical shunting procedures may be considered. Liver transplantation remains the definitive treatment for those with fulminant hepatic failure or disease unresponsive to prior interventions [19,20].

A recognized complication of ET is AvWS, which was present in our patient. The reported prevalence of AvWS in ET varies between 20% and 55% [21]. The condition arises from increased shear stress and heightened platelet-mediated adsorption and clearance of circulating vWF, resulting in impaired primary haemostasis and a heightened risk of bleeding [11,12]. This is of particular clinical significance in the context of portal hypertension, as illustrated in our patient who experienced recurrent variceal haemorrhages requiring multiple endoscopic band ligations despite initial stabilization. It is important to screen for AvWS in patients with ET, irrespective of platelet count. The underlying ET in this case was treated with cytoreductive agents to achieve disease remission.

## Conclusions

This case highlights the rare but important complication of BCS secondary to JAK2-positive ET. Early recognition and prompt treatment are critical for improving outcomes; however, diagnostic challenges arise when differentiating radiological features mimicking hepatic malignancy from thrombotic lesions. Clinicians should remain alert to the coexistence of AvWS in the setting of worsening thrombocytosis. Emphasizing the importance of systematically evaluating patients with ET for AvWS, given its significant therapeutic implications. Timely management with systemic anticoagulation and interventional procedures such as recanalization or venoplasty can improve prognosis. Balancing the competing risks of thrombosis and bleeding in ET requires tailored treatment strategies, including routine screening for AvWS and hepatic dysfunction. Ultimately, such complex cases are best addressed through a multidisciplinary team approach involving haematology, hepatology, and specialist coagulation services to provide comprehensive, evidence-based care.
